# Clinical reasoning in undergraduate paramedicine: utilisation of a script concordance test

**DOI:** 10.1186/s12909-023-04020-x

**Published:** 2023-01-19

**Authors:** Linda Ross, Eli Semaan, Cameron M. Gosling, Benjamin Fisk, Brendan Shannon

**Affiliations:** 1 Department of Paramedicine, School of Primary and Allied Health Care, Faculty of Medicine, Nursing and Health Science, Monash University, PO Box 527, Peninsula Campus, McMahons Road, Frankston, Melbourne, Victoria 3199 Australia; 2 Ambulance Victoria, Melbourne, Australia

**Keywords:** Paramedic, Paramedicine, Undergraduate, Clinical reasoning, Script concordance test, SCT

## Abstract

**Introduction:**

Clinical reasoning is a complex cognitive and metacognitive process paramount to patient care in paramedic practice. While universally recognised as an essential component of practice, clinical reasoning has been historically difficult to assess in health care professions. Is the Script Concordance Test (SCT) an achievable and reliable option to test clinical reasoning in undergraduate paramedic students?

**Methods:**

This was a single institution observational cohort study designed to use the SCT to measure clinical reasoning in paramedic students. Clinical vignettes were constructed across a range of concepts with varying shades of clinical ambiguity. A reference panel mean scores of the test were compared to that of students. Test responses were graded with the aggregate scoring method with scores awarded for both partially and fully correct responses.

**Results:**

Eighty-three student paramedic participants (mean age: 21.8 (3.5) years, 54 (65%) female, 27 (33%) male and 2 (2%) non-binary) completed the SCT. The difference between the reference group mean score of 80 (5) and student mean of score of 65.6 (8.4) was statistically significant (*p* < 0.001).

**Discussion:**

Clinical reasoning skills are not easily acquired as they are a culmination of education, experience and the ability to apply this in the context to a specific patient. The SCT has shown to be reliable and effective in measuring clinical reasoning in undergraduate paramedics as it has in other health professions such as nursing and medicine. More investigation is required to establish effective pedogeological techniques to optimise clinical reasoning in student and novice paramedics who are devoid of experience.

## Introduction

Clinical reasoning is a complex cognitive and metacognitive process [1] paramount to patient care in paramedic practice. Reasoning is a process that relates to thought processes, the arrangement of ideas, and assessment of experiences to reach conclusions [2]. In the clinical context paramedics draw on their specific scientific knowledge and clinical experience, and integrate that with what is known about the specific situation and patient. They must analyse and synthesis all this information and differentiate its usefulness and application to the patient. This process leads to an informed decision about patient management.

While universally recognised as an essential component of practice, clinical reasoning has been historically difficult to assess in health care professions. Various methods have been used to assess clinical reasoning with each having shortcomings. Multiple choice questions for example work in instances where there is a right answer, but are limited in conveying the inherit complexity, ambiguity and uncertainty of a clinical case [3]. Likewise, objective structured clinical examinations have been criticised for their drain on resources and lack of consistency between patients and assessors [4].

A diagnostic script questionnaire based on script theory was first developed by Charlin et al. in 1998 [5]. It was developed for use in health professions to assess clinical reasoning competence in uncertain circumstance [5]. It has since evolved into the Script Concordance Test (SCT) and is widely used across medicine [6, 7] nursing [8] and numerous allied health disciples [9]. It is a validated test and has been proven to have good internal consistency when development guidelines are followed [10, 11].

In the paramedicine context, clinicians regularly encounter complex patients with multiple comorbidities across the biopsychosocial spectrum [12, 13]. These episodes are often ill-defined with management not always fitting into a right or wrong category. It is therefore an important process for paramedics to develop clinical reasoning skills which incorporate their knowledge of pathologies and their previous experiences, in combination with the patient presentation, in order to determine treatment pathways. Paramedics also generally work in the out-of-hospital setting where episodes of care can occur in uncontrolled environments with time pressures [14]. These circumstances add additional cognitive load to clinicians trying to collect, process and make sense of complex information [13].

There is no published literature discussing the teaching and/or assessing of clinical reasoning in paramedicine despite its established importance. The SCT has been utilised in medicine and found to be a feasible option for assessing undergraduate medical students [15–17]. Likewise, in nursing, studies have found the SCT to be a reliable, standardised and easy to administer test to measure clinical reasoning [8, 18]. This study therefore aimed to determine if the SCT is an achievable and reliable option to test clinical reasoning in undergraduate paramedic students.

## Methods

This was a single institution observational cohort study conducted at an Australian university in August 2021. It was designed to use a previously validated tool, the Script Concordance Test (SCT), to measure clinical reasoning in third year undergraduate paramedic students. The study was approved by the Monash University Human Research Ethics Committee (MUHREC - 2021-29,344).

### Development of the script concordance test

Test construction occurred in accordance to SCT developmental guidelines published in an AMEE Guide by Lubarsky et al. [11]. The structure of the SCT aims to reflect the varying shades of clinical ambiguity inherit in real-world practice by presenting clinicians with a series of clinical vignettes with incomplete or ill-defined medical data. Development of the SCT was reliant upon three fundamental steps: creating clinical vignettes and test items formulated from actual cases, selection of the reference panel, and construction of the scoring matrix [11].

Test drafting required authors to create quality test questions representative of the paramedic discipline ensuring both content validity and clinical ambiguity. Clinical vignettes were constructed in alignment with the medical curriculum utilised by clinical faculty throughout the three-year undergraduate degree at Monash University. General medical domains including cardiac, respiratory, trauma, obstetric, and neurological pathologies were utilised, in addition to areas surrounding pharmacology and clinical practice guidelines. These domains are representative of the universal knowledge base students should be well versed in upon completion of the undergraduate degree. Clinical vignettes and questions were created based upon attracting both a wide range of responses from the available options and limiting the necessity for students to rely upon factual recall.

**Fig. 1:**
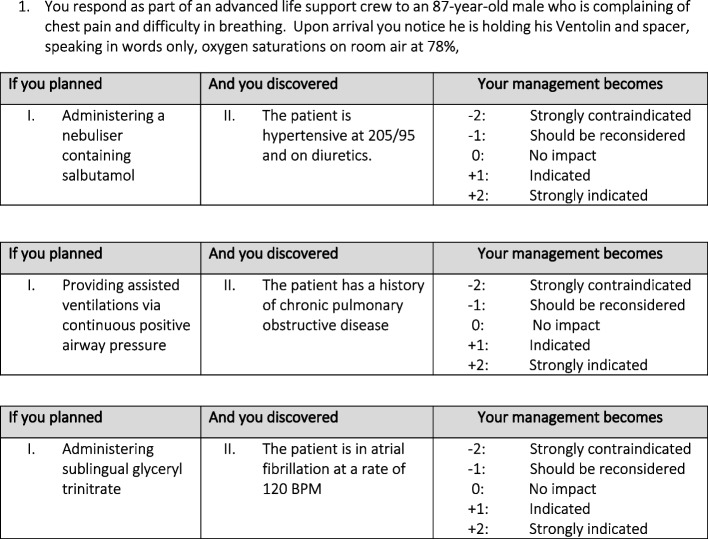
SCT Example

Three of the project team with vast clinical experience (BF, ES & BS) were utilised to verify the validity of the test aiming to ensure that scenarios were relevant to the paramedic discipline, required a degree of decision-making, and were correctly formatted. A fourth team member (LR) was responsible for proofreading test items prior to finalisation and dissemination of the test. Draft amendments occurred utilising collaborative software allowing for easy adjustments to occur amongst authors. A total of 11 clinical vignettes comprising of 28 questions were utilised, with the most frequent clinical questions exploring management and treatment modalities (*n* = 23), with the remaining questions related to patient assessment or disease pathology (*n* = 5). See Fig. 1 for example question and format.

### Reference panel selection

Developmental guidelines outline that 15 panel members are required for high stakes examinations, in addition to optimal panel composition requiring experts to be representative of the paramedic profession with sound clinical experience. Fifteen panel members were initially recruited, with one participant failing to complete the test. The final reference panel comprised of 14 experienced clinicians who are employed as operational paramedics with current registration and a minimum of 5 years’ experience. The reference panel consisted of Advanced Life Support (ALS) paramedics (*n* = 4), Intensive Care (IC) paramedics (*n* = 5) and Intensive Care Helicopter Emergency Medical Services (HEMS) paramedics (n = 5).

Identical tests were provided to the reference panel clinicians with the same test conditions and parameters. As the tailored SCT is characteristic of commonly encounter medical presentations, the reference panel clinicians completed the test without prior preparation.

### Scoring matrix

Test responses were graded in accordance with the literature by utilising the aggregate scoring method [11, 17, 19]. Contrary to traditional examinations which require test-takers to select the allocated single best answer, the SCT awards both partial and full credits to participant responses.

Scoring matrices are based upon the distribution amongst the reference panel for responses for each clinical question. Full credits are awarded to the modal answer (commonly chosen answer amongst the panel members); partial credits awarded to alternative answers dependent on the fraction of the reference panel selecting that response; remaining responses not chosen by the reference panel are awarded 0. Results are matched to explore the level of concordance between student and reference panel responses.

### Data collection

The SCT was introduced to students as part of a face-to-face tutorial sessions during week three of their final academic semester. They were provided with an introductory session aiming to familiarise participants with the structure and components of the SCT. Due to COVID restrictions at the time, a pre-recorded introduction by the chief researcher was provided to participants. Students were introduced to the objectives of the research, the importance of clinical reasoning within paramedic practice, and provided with an exemplar clinical vignette and questions.

### Data analysis

Data were reported using descriptive statistics including, means (standard deviations), medians (inter quartile ranges), ranges or frequencies where appropriate. Comparisons between the reference panel and student participants was evaluated using t-tests between mean scores. Standardisation of participant scores was completed using a standardisation method previously described by Piovezan et al. [17] and Charlin et al. [20]. One student response was excluded from analysis as a result of missing data, greater than 50% of responses. Internal consistency was evaluated and reported using Cronbach alpha. A post-test evaluation of the student experience using the SCT was reported as percentages who agreed or strongly agreed with statements about the test and process. Data were analysed using Stata version 15 (StataCorp, College Station, Texas) and statistical significance was assigned when *P* < 0.05.

## Results

The reference panel of 14 experienced paramedics comprising of nine male and five female ALS, IC and HEMS practitioners had a mean (SD) age of 37.9 (10.1) and 14.3 (8) years of experience. Eighty-two student paramedic participants (age: 21.8 (3.5) years, 54 (65%) female, 27 (33%) male and 2 (2%) non-binary) completed the SCT. Only seven (8%) students had previous clinical experience in various capacities including nursing, disability, pharmacy and dental.

The results of the comparison between the reference panel and student group are presented in Table 1. The difference between the reference panel mean score of 80 (5) and student mean of score of 65.6 (8.4) was statistically significant (*p* < 0.001). There was a greater variation in student scores compared to the reference panel group as demonstrated by a greater range of scores (Table 1). The internal consistency of the SCT was good with a Cronbach alpha of 0.79. After calculation of standardised scores for the student cohort additional comparisons demonstrated that the majority of students 59 (72%) were more than 2SD below the reference panel mean. Of the remainder nine (11%) students scored between 1 and 2 SDs below the panel mean, 12 (15%) scored between the mean and 1 SD below, and two (2%) students scored above the panel mean.

**Table 1:** SCT score comparison

	Mean (SD)	Median (IQR)	Minimum	Maximum	Range
Reference panel	80 (5)	79.7 (77.3–82.5)	72.5	87.7	15.1
Students	65.6 (8.4) *	65.8 (59.6–70.5)	42.3	86.2	43.8

* *P* < 0.001

Sixty-one percent of students found the SCT easy to use and was a useful fit for purpose tool. Students felt that they had sufficient time to undertake the test and 95% agreed that clinical reasoning should be taught and tested in undergraduate paramedicine degrees (Table 2).

**Table 2:** SCT student evaluation

ITEM	Agree or Strongly agree (%)
Well-defined & communicated	71%
Simple & easy to follow	61%
Sufficient time	90%
Accurately reflect clinical reasoning	59%
Taught & tested in UG	95%

## Discussion

Paramedics require a broad skill and knowledge base to enable correct identification of clinical priorities and the application of the correct medical interventions. It may be argued that clinical reasoning carries equal importance to practical skills and background medical knowledge. It follows that the development of clinical reasoning should commence during the early stages of paramedic education, with the intent that it will further develop as clinical exposure and experience is gained. In order to teach clinical reasoning, it first needs to be defined and measured. This can be challenging, and to the authors’ knowledge, clinical reasoning has not previously been measured in a student paramedic cohort. The results of this study have shown the Script Concordance Test (SCT) to be a reliable and effective method to compare clinical reasoning between undergraduate student paramedics and experienced registered paramedics. A statistically significant difference in the mean SCT score (*p* < 0.001) was found between the reference panel and student cohorts. The internal consistency of the SCT was supported by a Cronbach alpha score of 0.79, indicating good reliability for the use of the SCT within the paramedic groups.

The SCT has previously been shown to be a valid test for clinical reasoning in many health professions [21]. Paramedicine is an evolving and increasingly professionalised health discipline and the development of clinical reasoning is one of, if not the most important factor for achieving positive patient outcomes. Paramedics routinely work in limited resource and chaotic environments and many decisions regarding, in some instances, quite complex interventions, need to be made quickly and under high stress [22]. To this end the development of student paramedics to be clinically prepared at the end of their formal academic education continues to be a challenge for educators [23]. Results from this study have provided valuable insight into the validity of the SCT in paramedicine and the variance between expert and novice paramedics clinical reasoning. The mean result clearly shows that expert paramedics included in this study demonstrated better clinical reasoning. This is not surprising and is consistent with previous literature pertaining to other healthcare professions [24].

The variance in the student paramedic results compared to the expert paramedic group highlight some other interesting points. While the mean score was 14.4 points below the mean reference panel score, 71% of student responses were two standard deviations below the reference panel mean score. This truly identifies the gap between expert and student and highlights the need for greater emphasis in educational programs being directed towards developing clinical reasoning skills. Clinical reasoning skills have been reported to develop over time and with experience, [25] however, targeted education with clinical application in simulated clinical environments can increase clinical reasoning ability in novices [26]. It is noted that the need for clinical reasoning to be integrated into medical courses throughout each year is an important curriculum development area and paramedicine should be no different [27]. Integration of clinical reasoning teaching throughout each year of paramedicine courses would ensure students are better prepared for the clinical reasoning requirements of clinical practice on commencement of their clinical practice.

While most students fell below the reference panel mean there were exceptions with two students scoring above the panel mean. An investigation into these outliers may reveal certain experiential or academic characteristics that are linked to the positive result and would assist in understanding what contributes to expert level clinical reasoning ability at this level. Conversely, as the range of results show the lowest standardised score recorded in the student group was 42.3, almost 40 points below the reference panel group and their highest scoring counterparts. An investigation into the lower range of scores may reveal characteristics that are linked to poor clinical reasoning ability at this level. The SCT should not however be used solely to evaluate clinical reasoning [28]. The use of the SCT to identify students requiring further support in clinical reasoning where possible is something to be considered. This process would allow educators to implement educational opportunities in order to make student paramedics as prepared as possible for the clinical reasoning requirements associated with safe clinical practice. Previous literature has supported the use of the SCT as a tool to identify and support physicians requiring additional support in clinical reasoning or at a minimum allows recognition and acknowledgement of gaps in clinical reasoning [7, 28].

In the participant evaluation of the SCT completed by the novice group, 59% of the paramedic students felt that the questions in the SCT accurately reflected clinical reasoning. Similarly, 61% of the student group felt that the SCT was simple and easy to follow. An evaluation of the SCT was not completed by the reference panel, so a direct comparison cannot be made. The student evaluation may in fact support the notion that an understanding of clinical reasoning as a concept and a process develops over time [29]. The questions in the SCT needed a degree of ambiguity to allow clinical reasoning to be applied, and the results of the student evaluation may indicate that ambiguity in clinical problem solving can be challenging for novice clinicians [30].

## Limitations

One of the limitations of this study was that only one-year level was used to compare novices with the reference panel. A comparison of first and second year paramedic student results in addition to third and final years may have shown a developmental increase in clinical reasoning skills over time. The selection of final year paramedicine students was based on the rational they would have the requisite background clinical knowledge (being in the final semester of their degree) and have most placement experience completed which in combination would enable them to answer the SCT questions. This study was conducted during the COVID-19 pandemic in an environment of reduced face to face clinical simulations and clinical placements. It is possible that this could have impacted the development of student clinical reasoning ability. Finally, previous studies have questioned the utility of the SCT for use in high stakes assessments [31, 32]. However, data in this study indicate the test was reliable, had face validity, was effective and yielded similar results to other student populations when used as a formative tool as part of a wide-ranging approach to assessing clinical reasoning. To determine if the SCT is a truly valid test of clinical reasoning for paramedic student populations further validity and scoring testing would be required.

## Conclusion

Clinical reasoning prowess is not easily acquired as it is the culmination of education, experience and the ability to apply this in the context of a specific patient and situation. The importance of clinical reasoning in the out-of-hospital environment can not be underestimated as paramedics must make safe and timely clinical decisions without support mechanisms otherwise available in a hospital setting. The SCT has shown to be a reliable and effective measure of clinical reasoning in undergraduate paramedics. More investigation is required to establish effective pedogeological techniques which optimise clinical reasoning in student and novice paramedics who are devoid of the experience which can only be gained over time.

## Data Availability

The datasets used and/or analysed during the current study available from the corresponding author on reasonable request.
